# Supplementary material for: Orbital Polarization-Dependent Fragment Twist-Induced Intramolecular Electric-Field-Driven Charge Transfer
Source: Molecules. 2023 Feb 14;28(4):1801. doi: 10.3390/molecules28041801 (PMC9961529; doi:10.3390/molecules28041801)
Supplement: Supplementary file 1 [file molecules-28-01801-s001.zip › molecules-2178809-supplementary.pdf]

Support information

Orbital polarization dependent fragment twist-induced intra-molecular electric field driven charge transfer

Wenjing Bo [1], Hao Sheng [1,*] and Jingang Wang [1,*]

S1. Method

S1.1. Dipole moments

Dipole moments are vital for analyzing local polarities. In this section, the dipole moment is obtained by quantum chemical. The formula for calculating the dipole moment vector of a fragment F composed of certain atoms in the system is (S1):

$$\mathbf{D}_F = \sum_{A \in F} \left[Z_A R_A - \int w_A(\mathbf{r}) \rho(\mathbf{r}) \mathbf{r} d\mathbf{r} \right] \tag{S1}$$

Where R is the nuclear position, Z is the nuclear charge, ρ is the electron density, and w is the atomic weight function.

S1.2. Electrostatic potential

Electrostatic potential refers to the electrostatic interaction between a unit point charge at the r position and the system. The formula for calculating the electrostatic potential in a molecular system is (S2):

$$V_{\mathbf{Total}}(\mathbf{r}) = V_{\mathbf{Nuc}}(\mathbf{r}) + V_{\mathbf{Elec}}(\mathbf{r}) = \sum_A \frac{Z_A}{|\mathbf{r} - R_A|} - \int \frac{\rho(\mathbf{r}')}{|\mathbf{r} - \mathbf{r}'|} d\mathbf{r}' \tag{S2}$$

where Z and R represent the nuclear charge and nuclear position, respectively. Positive (negative) values indicate positions governed by nuclear (electron) charge.

S1.3. Polarity

To quantify molecular polarity, a molecular polarity index (MPI) [43-44] is defined, which is related to the Π index. The calculation formula of (MPI) is (S3):

$$\mathrm{MPI} = (1/\mathrm{t}) \sum_{\mathrm{k}=1}^{\mathrm{t}} |\mathrm{V}(\mathbf{r}_\mathrm{k})| \equiv (1/\mathrm{A}) \iint_\mathrm{S} |\mathrm{V}(\mathbf{r})| \mathrm{dS} \tag{S3}$$

where V is the molecular electrostatic potential, the integral is the integral over the molecular surface S, and A is the molecular surface area, and Π is the average deviation across the surface, regarded as an indicator of internal charge separation. MPI is a very reliable norm of molecular polarity, and the larger the index, the greater the polarity.

S1.4. Density of states

When calculating the DOS in quantum chemistry, the original total DOS (TDOS) of the isolated system can be written as (S4)

$$TDOS(E) = \sum_i \delta(E - \varepsilon_i) \tag{S4}$$

where $\{\varepsilon\}$ is the orbital energy and δ_{iS} is the Dirac function.

The TDOS value has the same interval at each molecular orbital energy level point, and the TDOS plot is drawn as a series of vertical lines. The DOS curve cannot clearly show the density of states of the separated system, and the orbital distribution density of different energy regions cannot be analyzed intuitively, so the broadening function $F(x)$ is used to replace δ_{iS}, and the broadened TDOS is obtained. The normalized Gaussian function is defined as:

$$G(x)=\frac{1}{c\sqrt{2\pi}}e^{-\frac{x^2}{2x^2}} \tag{S5}$$

$$c=\frac{FWHM}{2\sqrt{2\ln 2}} \tag{S6}$$

The larger the full width at half maximum (FWHM), the larger the peak of the curve, the smoother the DOS graph looks, and the more helpful for research. The curve that a specific segment contributes to TDOS is the local DOS. By defining the segment, this work studies the nature and bulk composition of orbitals in different energy intervals in the PDOS diagram. For example, define the PDOS of fragment A as formula (S7) $\Theta_{i,A}$ where is the composition of fragment A in orbit i.

$$PDOS_A(E)=\sum_i \Theta_{i,A}F(E-\varepsilon_i) \tag{S7}$$

For such close structures, they have similar energies and roughly the same number of states. In the energy level range from -21.7 to -5.3 eV, the total density of states is dominated by the PX, PY and PZ orbitals. In the energy level range from −2.2 to 5.5 eV, the overall density of states is dominated by the PZ orbitals, which allows more electrons to remain in the PZ orbitals and be spin-polarized, which in turn enhances the local spin-polarization. The DOS between the two spikes flanking the Fermi level of O9H in the six figures is not zero and is broad.It shows that the covalent bond of the system is relatively strong at this time, and the smaller the electronegativity, the greater the polarization.

S1.5. Electrons and holes

The optical properties of molecules largely depend on the transition of electrons. According to the quantum theory of electronic transitions, the transition probability is related to the intrinsic dipole integral of the ground and excited states. The dipole moment can be seen as the electric dipole generating a local electric field so the modulation of the intramolecular electric field can affect the electronic transition. Expressions for electrons and holes for TDHF/TDDFT are (S8):

$$\begin{aligned}
&\rho^{\text{hole}}(\mathbf{r})=\rho^{\text{hole}}_{(\text{loc})}(\mathbf{r})+\rho^{\text{hole}}_{(\text{cross})}(\mathbf{r})\\
&\rho^{\text{hole}}_{(\text{loc})}(\mathbf{r})=\sum_{i\to a}\left(w_i^a\right)^2\varphi_i\varphi_i-\sum_{i\leftarrow a}\left(w_i^{'a}\right)^2\varphi_i\varphi_i\\
&\rho^{\text{hole}}_{(\text{cross})}(\mathbf{r})=\sum_{i\to a}\sum_{j\neq i\to a}w_i^a w_j^a\varphi_i\varphi_j-\sum_{i\leftarrow a}\sum_{j\neq i\leftarrow a}w_i^{'a}w_j^{'a}\varphi_i\varphi_j\\
&\rho^{\text{ele}}(\mathbf{r})=\rho^{\text{ele}}_{(\text{loc})}(\mathbf{r})+\rho^{\text{ele}}_{(\text{cross})}(\mathbf{r})\\
&\rho^{\text{ele}}_{(\text{loc})}(\mathbf{r})=\sum_{i\to a}\left(w_i^a\right)^2\varphi_a\varphi_a-\sum_{i\leftarrow a}\left(w_i^{'a}\right)^2\varphi_a\varphi_a\\
&\rho^{\text{ele}}_{(\text{cross})}(\mathbf{r})=\sum_{i\to a}\sum_{i\to b\neq a}w_i^a w_i^a\varphi_a\varphi_b-\sum_{i\leftarrow a}\sum_{i\leftarrow b\neq a}w_i^{'a}w_i^{'a}\varphi_a\varphi_b
\end{aligned} \tag{S8}$$

Where $\mathbf{r}$ is the coordinate vector, φ is the orbital wave function, i or j is the occupied orbital label, and a or b is the empty orbital label. $\sum i \rightarrow a$ represents the excited configuration of the cycle, and $\sum i \leftarrow a$ represents the de-excited configuration of the cycle. The distribution of electrons and holes has local terms and cross terms. The dominant term is about the local term, which reflects the contribution of the configuration function, while the cross term can be ignored because it reflects the effect of the coupling between the configuration functions on the distribution of holes and electrons. TDM was used to analyze the photoelectron excitation properties.The transition density matrix is an effective tool for analyzing charge transfer between atoms.The core part of the transfer density matrix is to obtain the transfer density. It can be written in the basis function as (S9):

$$P_{\mu\nu}^{tran} = \sum_{i}^{occ} \sum_{j}^{vir} w_i^j c_{\mu i} c_{\nu j} \tag{S9}$$

where $C_{\mu i}$ represents the expansion coefficient of the basis function μ in molecular orbital i. And TDM in real space can be easily constructed by TDM in basis function representation.

$$T(\mathbf{r};\mathbf{r}') = \sum_{\mu} \sum_{\nu} P_{\mu\nu}^{tran} \chi_{\mu}(\mathbf{r}) \chi_{\nu}(\mathbf{r}') \tag{S10}$$

The off-diagonal elements of the TDM essentially represent the coupling between various basis functions during electron excitation. The excitation can be perfectly expressed as an orbital transition, and the TDM can be written explicitly as (S11):

$$P_{\mu\nu}^{tran} = \begin{bmatrix} C_{\nu i} C_{\mu j} & C_{\nu i} C_{\nu j} \\ C_{\mu i} C_{\mu j} & C_{\mu i} C_{\nu j} \end{bmatrix} \tag{S11}$$

If the off-diagonal elements are large, this means that the basis functions contribute significantly to holes (occupant orbitals) and electrons (virtual orbitals), respectively. In this case, it can be said that the two basis set functions are strongly coupled in the excitation process. Since the transition density is contributed by different basis functions (those belonging to different atomic centers), the transitions represented by this transition density can be regarded as charge transfer excitations. The diagonal term also makes sense if the element has a larger magnitude, which means that the basis function must have a larger contribution to both holes and electrons. First of all, according to the numerical value of the elements on the diagonal, it can be judged where the electrons and holes have a significant distribution at the same time. For the electron-hole density map, the blue and red isosurfaces describe the electron and hole densities, respectively. In the TDM diagram, the diagonal part represents the local excitation and the off-diagonal part represents the charge transfer. The abscissa and ordinate correspond to the positions of holes and electrons, respectively. The position of electron-hole density change can intuitively reflect the increase and decrease regions of electron density in the transition of electrons from the ground state to the excited state, and can help to understand the internal characteristics of electronic excitation.

In this work, electron excitation characteristics are discussed by some quantitative numerical methods：

$$S_{\mathrm{r}}\ \text{index}=\int S_{\mathrm{r}}(\mathbf{r})d\mathbf{r}\equiv\int \min\left[\rho^{\text{hole}}(\mathbf{r}),\rho^{\text{ele}}(\mathbf{r})\right]d\mathbf{r}$$
$$S_{\mathrm{r}}\ \text{index}=\int S_{\mathrm{r}}(\mathbf{r})d\mathbf{r}\equiv\int \sqrt{\rho^{\text{hole}}(\mathbf{r})\rho^{\text{ele}}(\mathbf{r})}d\mathbf{r}$$
$$D\ \text{index}=\sqrt{(D_{\mathrm{x}})^2+(D_{\mathrm{y}})^2+(D_{\mathrm{z}})^2}$$
$$\sigma_{\text{hole,x}}=\sqrt{\int(x-X_{\text{hole}})^2\rho^{\text{hole}}(\mathbf{r})d\mathbf{r}}$$
$$\Delta\sigma\ \text{index}=|\sigma_{\text{ele}}|-|\sigma_{\text{hole}}| \tag{S12}$$
$$H_{\lambda}=(\sigma_{\text{ele},\ \lambda}+\sigma_{\text{hole},\ \lambda})/2 \qquad \lambda=\{x,y,z\}$$
$$H_{CT}=H$$
$$H\ \text{index}=(|\sigma_{\text{ele}}|+|\sigma_{\text{hole}}|)/2$$
$$t\ \text{index}=D\ \text{index}-H_{CT}$$

S1.6. The transition dipole moment

The matrix element of the transition dipole moment matrix is defined as the product of the dipole moment integral between the corresponding TDM matrix element and the corresponding basis function. The transition dipole moment matrix has three components, X , Y , and Z, such as the sum of all matrix elements of the X component matrix is exactly the X component of the system transition dipole moment. The transition dipole moment matrix can also be contracted into atom-based form, and the X, Y , and Z components of the atomic transition dipole moment matrix are defined as (S13):

$$D_{A,B}^{X}=\sum_{\mu\in A}\sum_{\nu\in B}P_{\mu\nu}^{\text{tran}}\langle\chi_{\mu}|-x|\chi_{\mu}\rangle$$
$$D_{A,B}^{Y}=\sum_{\mu\in A}\sum_{\nu\in B}P_{\mu\nu}^{\text{tran}}\langle\chi_{\mu}|-y|\chi_{\mu}\rangle \tag{S13}$$
$$D_{A,B}^{Z}=\sum_{\mu\in A}\sum_{\nu\in B}P_{\mu\nu}^{\text{tran}}\langle\chi_{\mu}|-z|\chi_{\mu}\rangle$$

S2. Figures, Tables

Table S1. The dipole moments and dipole moment components of benzo [ghi] perylene of the compound, the side and the whole system are shown in the table.

a.u.		dx	dy	dz	total
O7H	all	-0.0308	0.0057	-0.0007	0.0313
	benzo[ghi]perylene	0.5891	0.0418	-0.0958	0.5983
	side	-0.6199	-0.0361	0.0951	0.6282
O8H	all	-0.0768	0.0205	-0.0188	0.0817
	benzo[ghi]perylene	0.3668	0.5301	-0.1215	0.6560
	side	-0.6588	0.0993	0.0101	0.6663
O9H	all	-0.1120	-0.0452	0.0060	0.1210
	benzo[ghi]perylene	0.7325	-0.1669	-0.1155	0.7601

	side	-0.8445	-0.1217	-0.1214	0.8618
OO7H	all	0.0065	-0.0526	0.0000	0.0530
	benzo[ghi]perylene	-0.9407	-0.0832	0.0000	0.9444
	side	0.9472	0.0307	0.0000	0.9477
OO8H	all	-0.0854	0.1855	-0.0207	0.2053
	benzo[ghi]perylene	0.9260	-0.2701	-0.0807	0.9680
	side	-0.9851	0.1882	0.0107	1.0030
OO9H	all	-0.0410	0.0182	0.0038	0.0450
	benzo[ghi]perylene	-2.0082	0.7801	-0.2596	2.1699
	side	-1.3918	0.2977	0.1819	1.4349

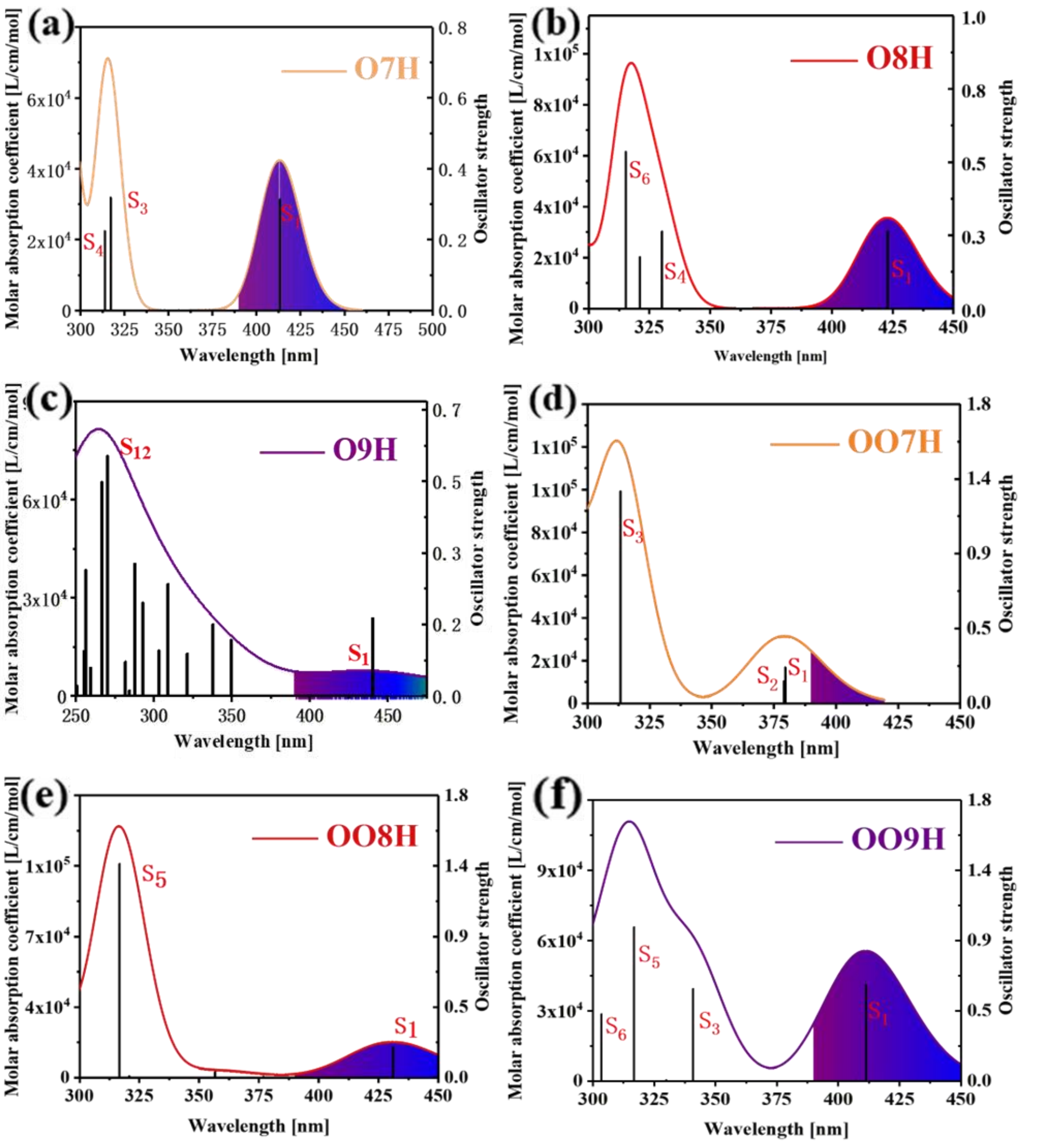

Figure S1. UV-Vis absorption spectra of 6 molecules: (a) O7H, (b) O8H, (c) O9H, (d) OO7H, (e) OO8H, (f) OO9H.

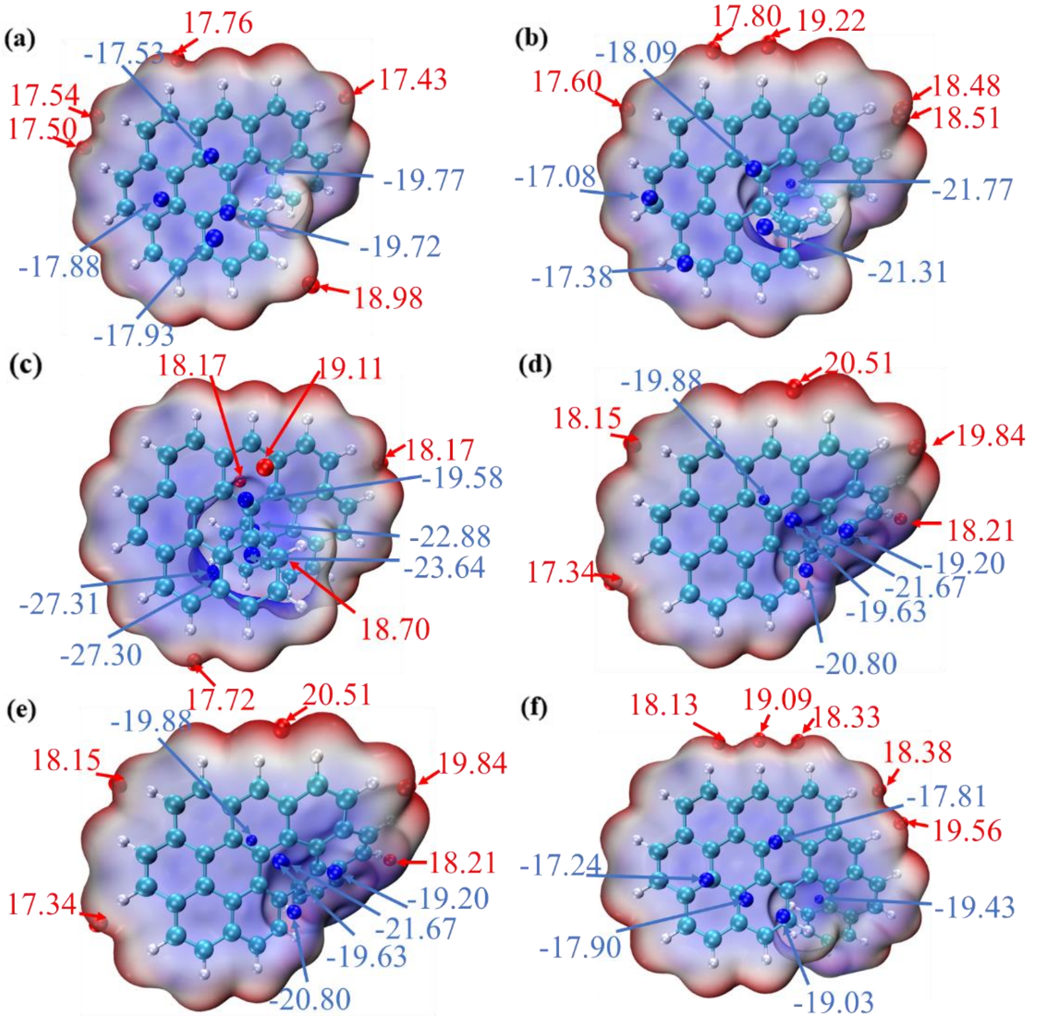

Figure S2. Electrostatic potential map of 6 molecules: （a） O7H, （b） O8H, （c） O9H, （d） OO7H, （e） OO8H, （f） OO9H.

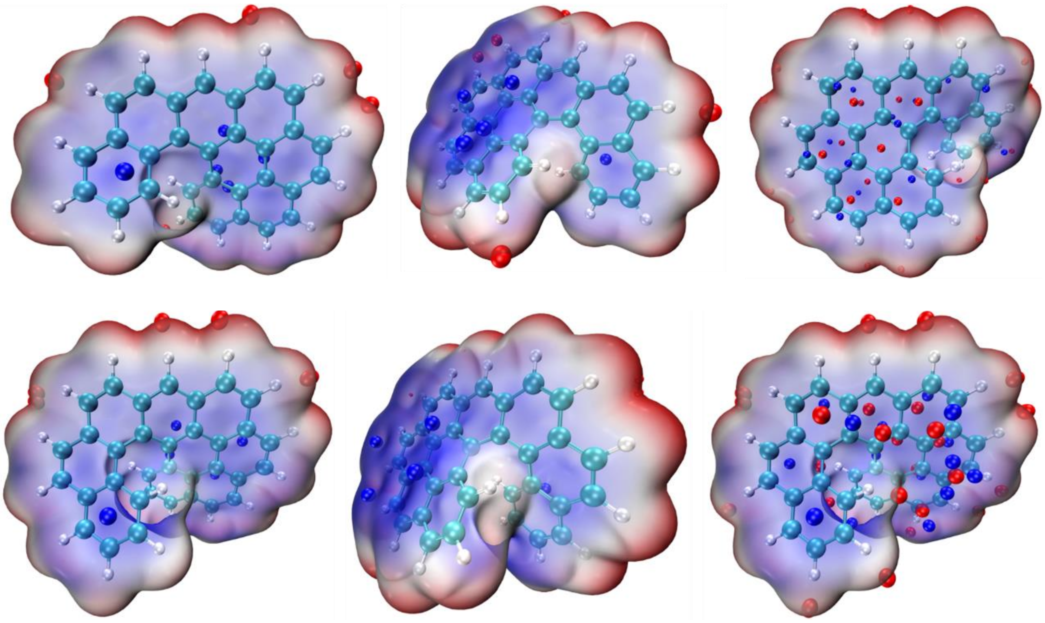

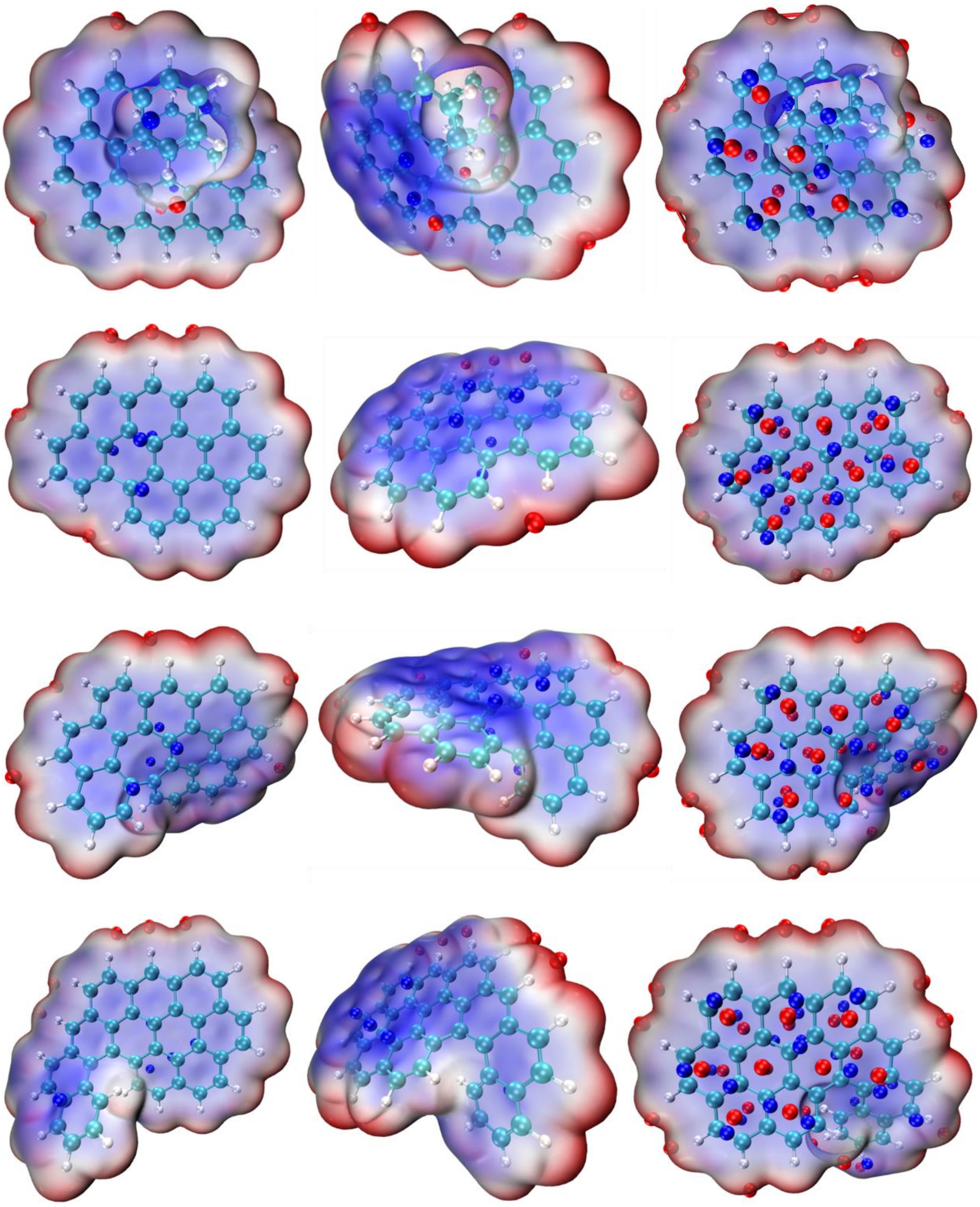

Figure S3. Electrostatic potential of 6 molecules from multiple angles.

Table S2. MPI values for 6 molecules.

Molecular	MPI(kcal/mol)
O7H	10.41235
O8H	10.57574
O9H	10.60316
OO7H	11.49283
OO8H	11.48095
OO9H	10.79676

Table S3. Energy level and energy gap difference of 6-molecule HOMO-LUMO.

Molecular	E_{HOMO}(eV)	E_{LUMO}(eV)	ΔE_{gap}(eV)
O7H	-6.324892	-1.190331	5.134561
O8H	-6.286601	-1.243465	5.043136
O9H	-6.249785	-1.261893	4.987892
OO7H	-6.345477	-1.119999	5.235478
OO8H	-6.145297	-1.165988	4.979309
OO9H	-6.263892	-1.285898	4.977995

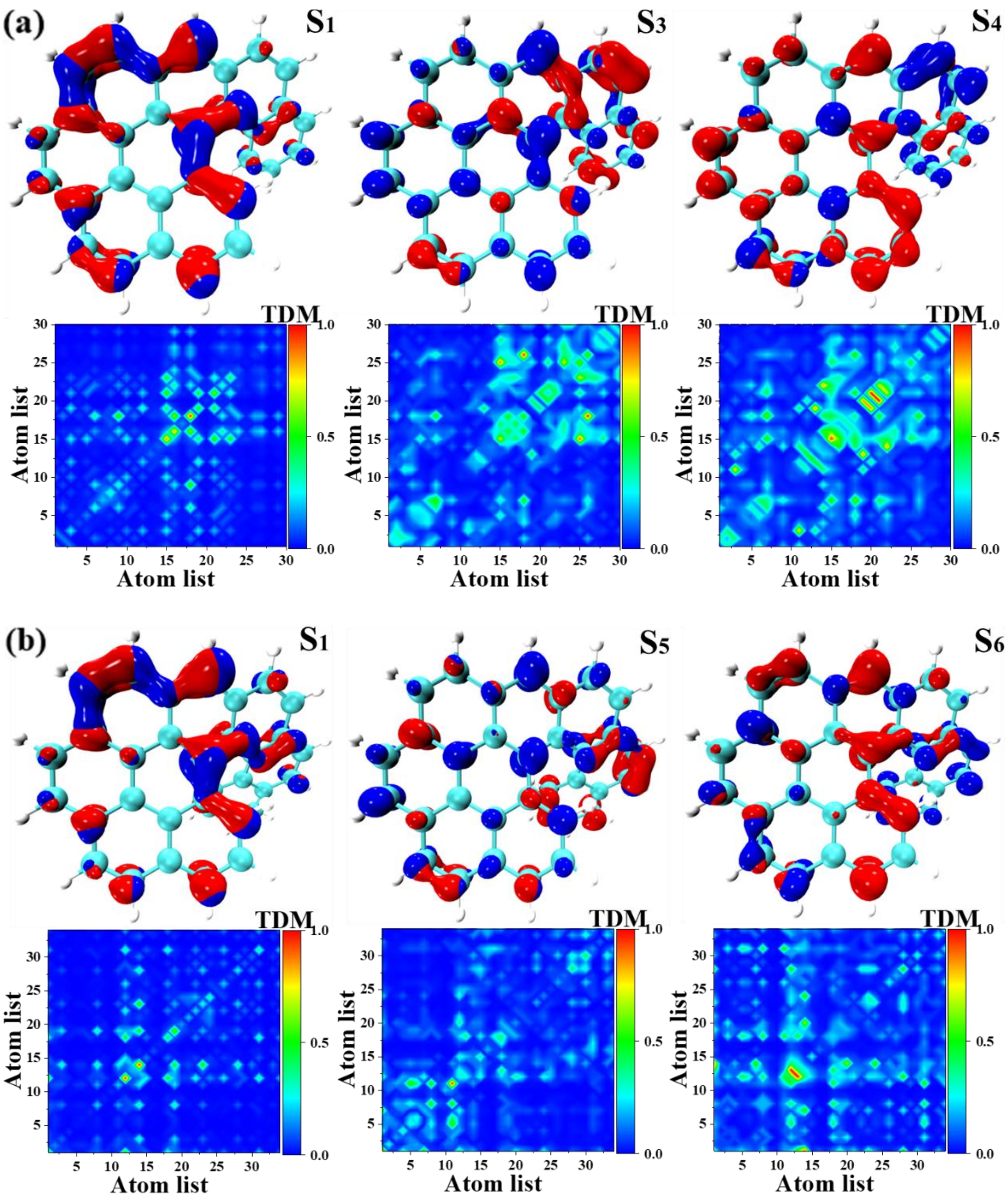

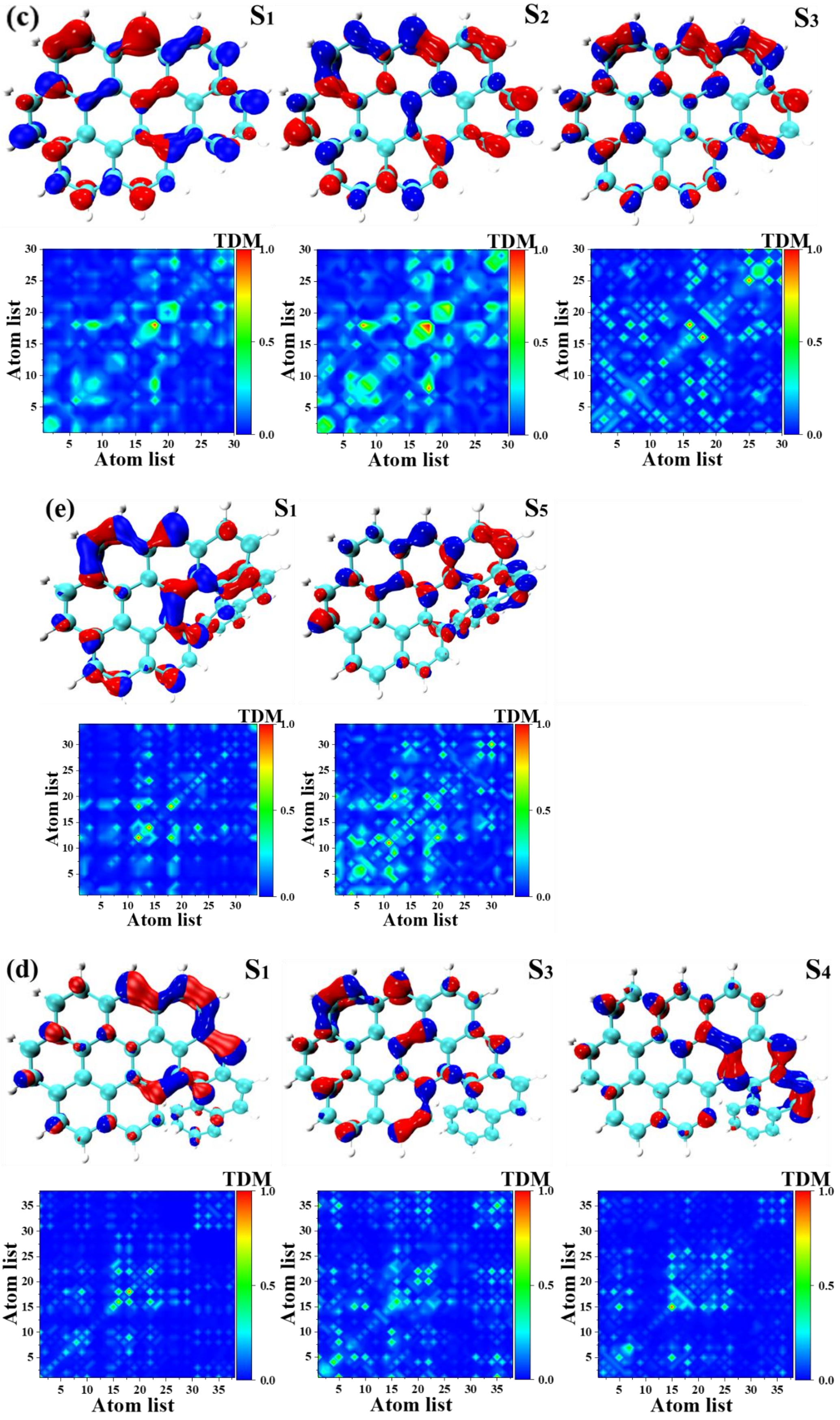

Figure S4. Electron-hole density map and transition density matrix of (a) O7H, (b) O8H, (c) OO7H, (e) OO8H, (d) OO9H.

Table S4. Electronic excitation characteristic parameters of the main excited states of the six molecules.

O7H	S1	S3	S4
Sm	0.67506	0.58833	0.62041
Sr	0.87763	0.86451	0.88506
Δσ	-0.003	-0.083	0.141
H index	3.5852	3.699	3.711
D index	0.169	1.089	0.966
t index	-2.347	-1.787	-1.843
Excitation energy (eV)	3.001	3.913	3.954

O8H	S1	S5	S6
Sm	0.68206	0.61974	0.6109
Sr	0.88224	0.87835	0.87775
Δσ	0.041	-0.061	0.347
H index	3.656	3.892	3.83
D index	0.195	1.019	0.676
t index	-2.412	-1.743	-1.969
Excitation energy (eV)	2.933	3.936	4.188

O9H	S1	S12
Sm	0.65921	0.68605
Sr	0.86917	0.91584
Δσ	0.042	-0.186
H index	3.607	3.739
D index	0.263	0.115
t index	-2.217	-1.854
Excitation energy (eV)	2.816	4.589

OO7H	S1	S2	S3
Sm	0.66711	0.68402	0.77925
Sr	0.9081	0.91657	0.9447
Δσ	0.113	-0.08	0.003
H index	3.864	3.834	3.886
D index	0.323	0.297	0.04
t index	-2.459	-2.448	-2.772
Excitation energy (eV)	3.269	3.274	3.961

OO8H	S1	S5
Sm	0.60692	0.72533
Sr	0.83661	0.92942
Δσ	-0.21	0.019
H index	3.727	3.945
D index	0.547	0.214
t index	-2.071	-1.93

Excitation energy (eV)	2.879	3.917

OO9H	S1	S3	S5	S6
Sm	0.69532	0.74232	0.73626	0.73654
Sr	0.89955	0.92729	0.92762	0.92858
$\Delta\sigma$	0.031	0.043	-0.071	-0.093
H index	4.11	4.08	4.178	4.138
D index	0.187	0.176	0.139	0.056
t index	-2.875	-2.76	-2.813	-1.312
Excitation energy(eV)	3.016	3.64	3.917	4.088

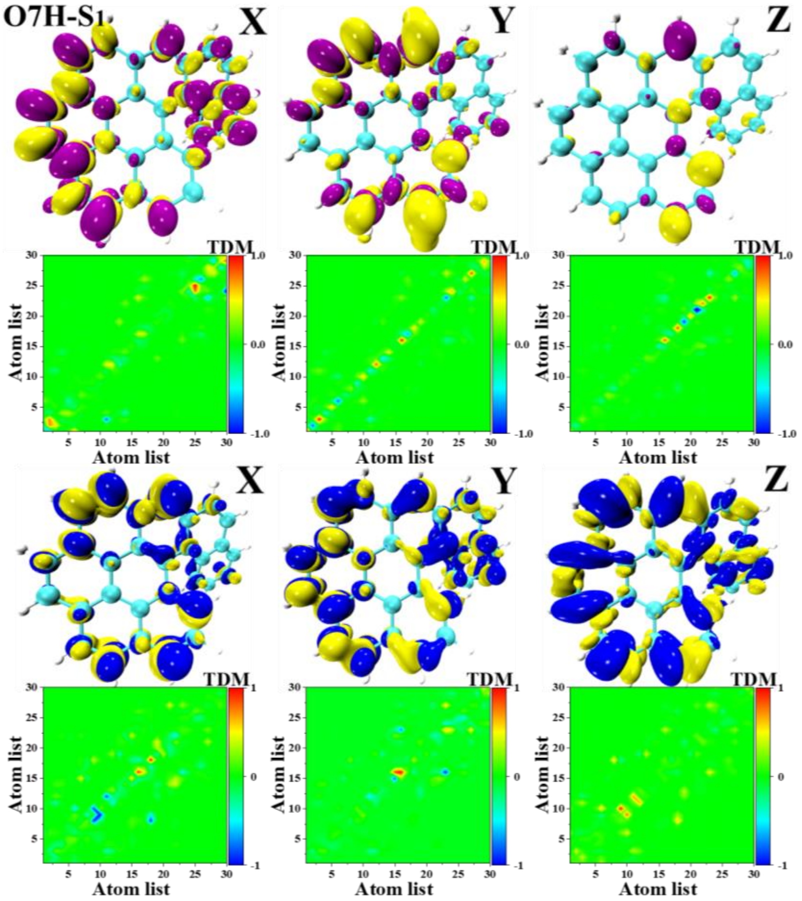

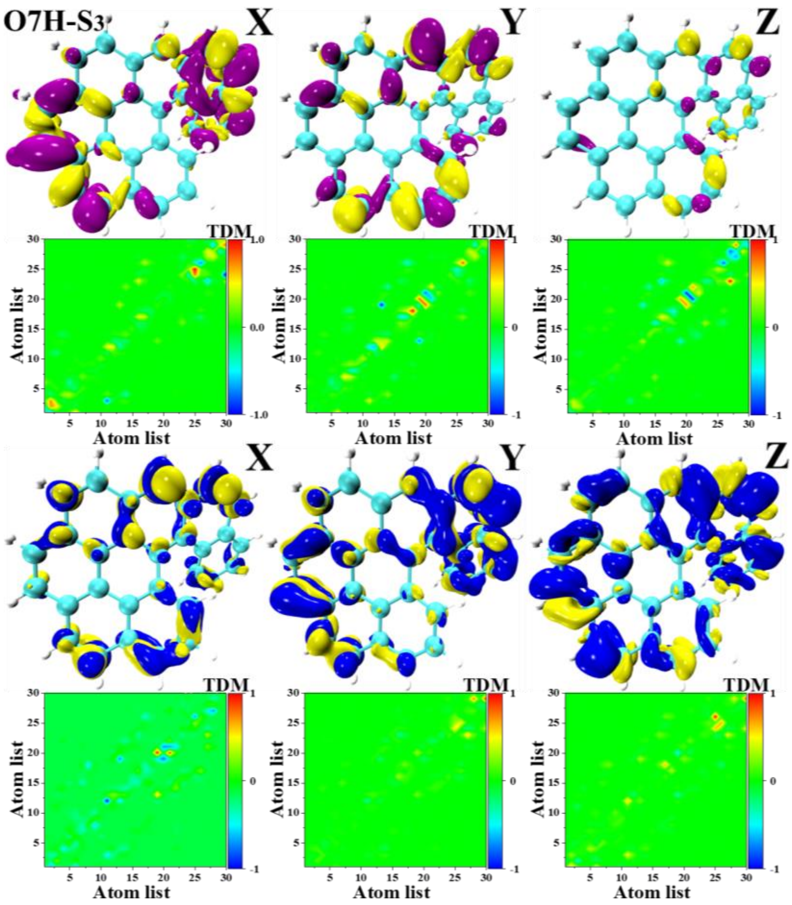
O7H-S3
X
Y
Z
TDM
Atom list
Atom list

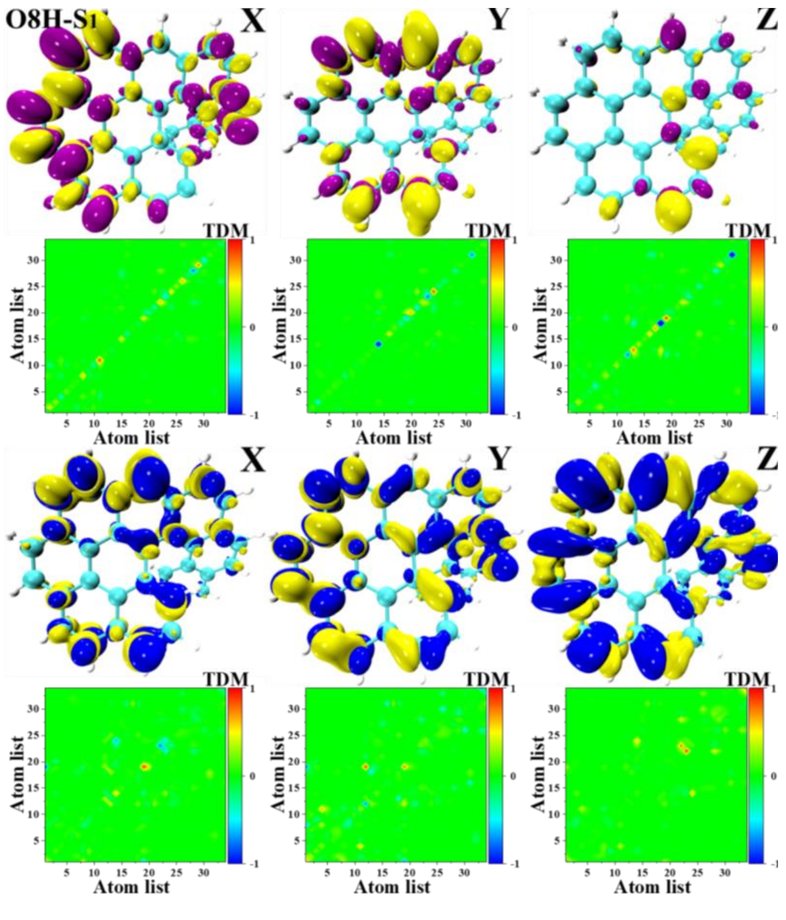
O8H-S1
X
Y
Z
TDM
Atom list
Atom list

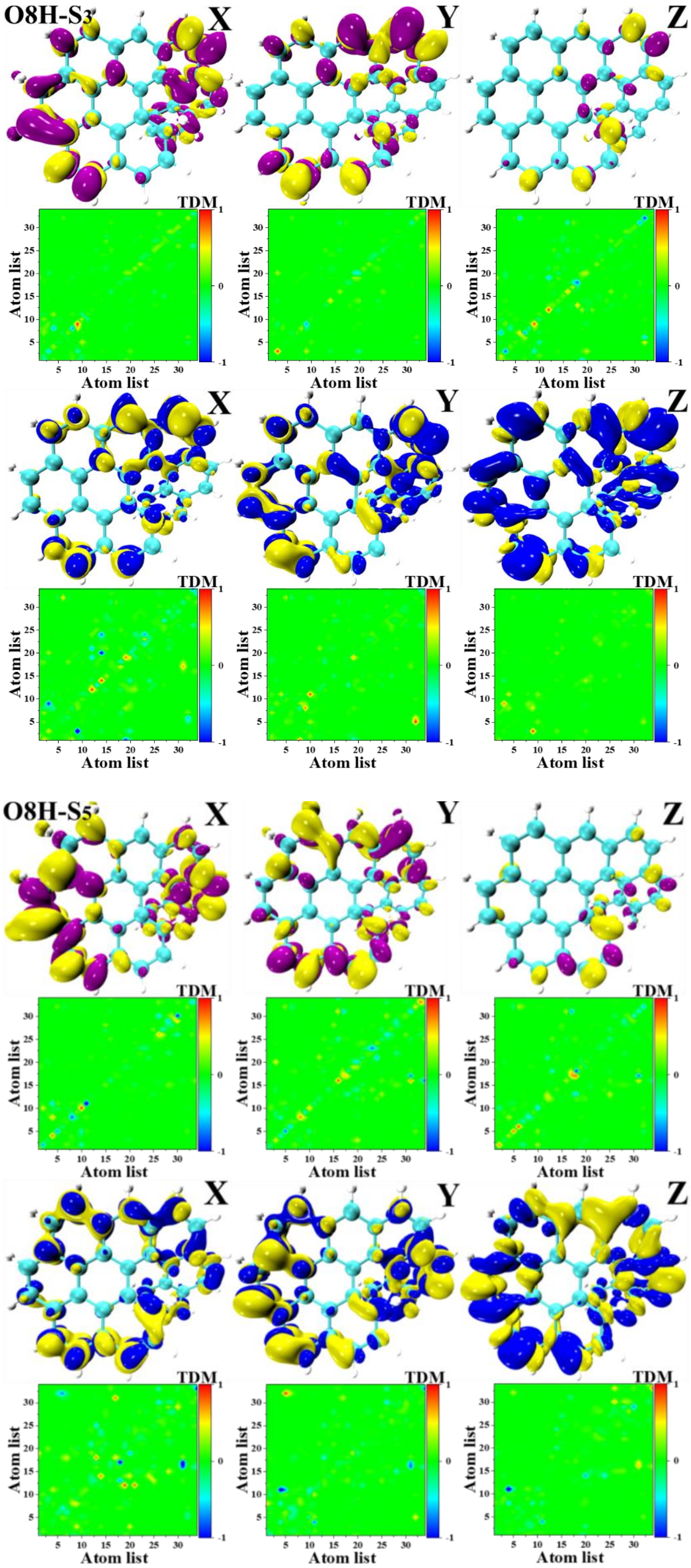
O8H-S3
X
Y
Z
TDM
Atom list
O8H-S5

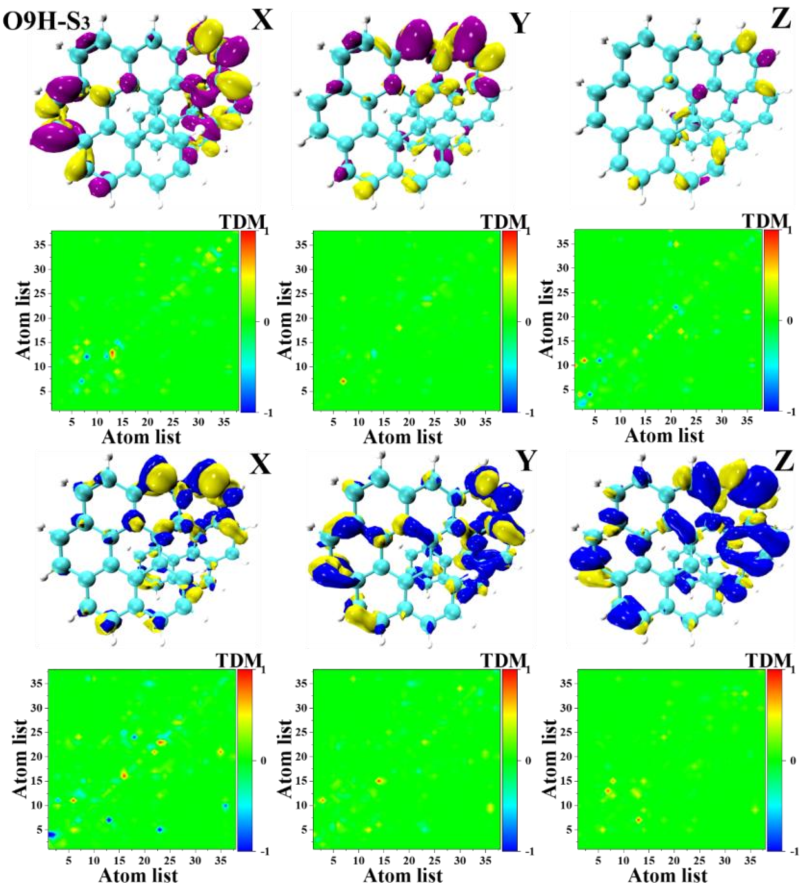
O9H-S3
X
Y
Z
TDM
Atom list

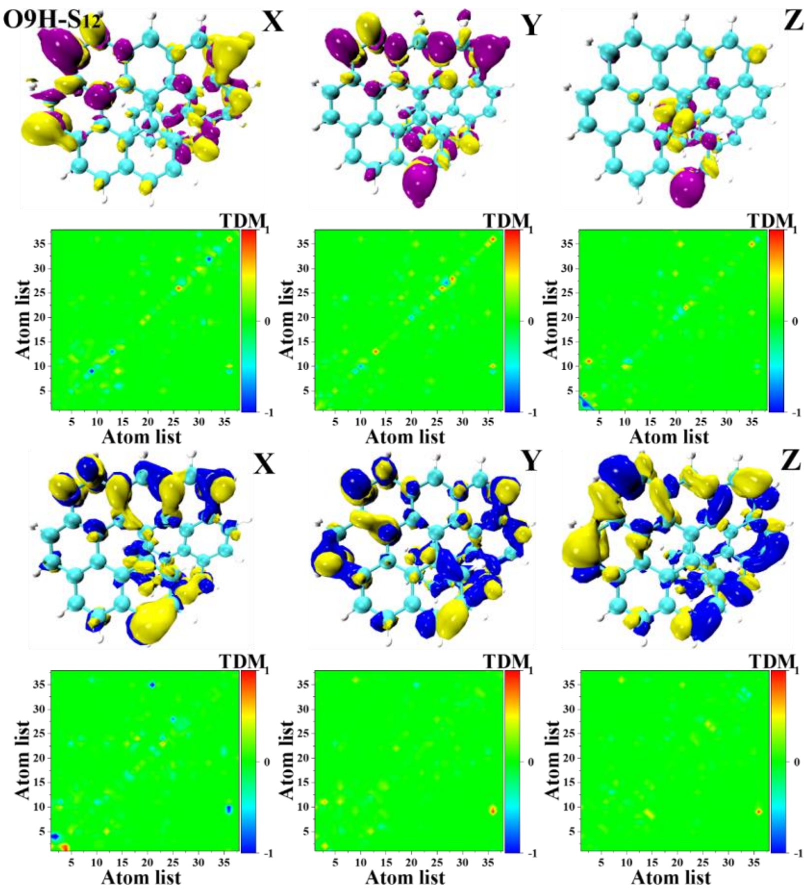
O9H-S12
X
Y
Z
TDM
Atom list

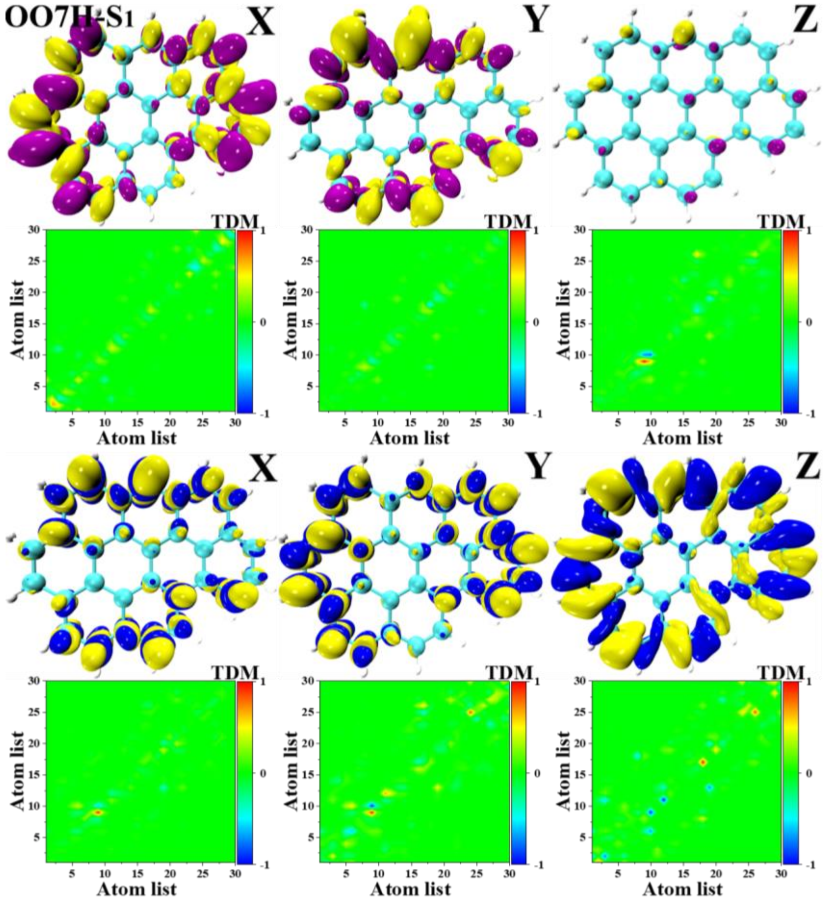
OO7H-S1
X
Y
Z
TDM
Atom list
Atom list

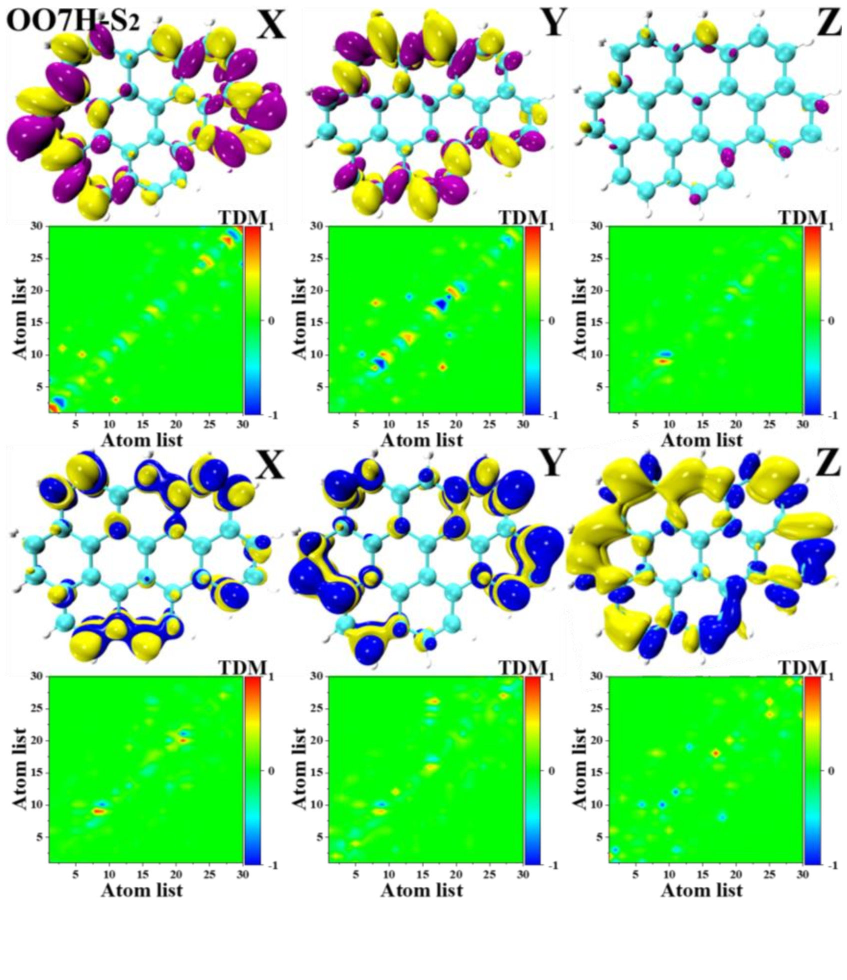
OO7H-S2
X
Y
Z
TDM
Atom list
Atom list

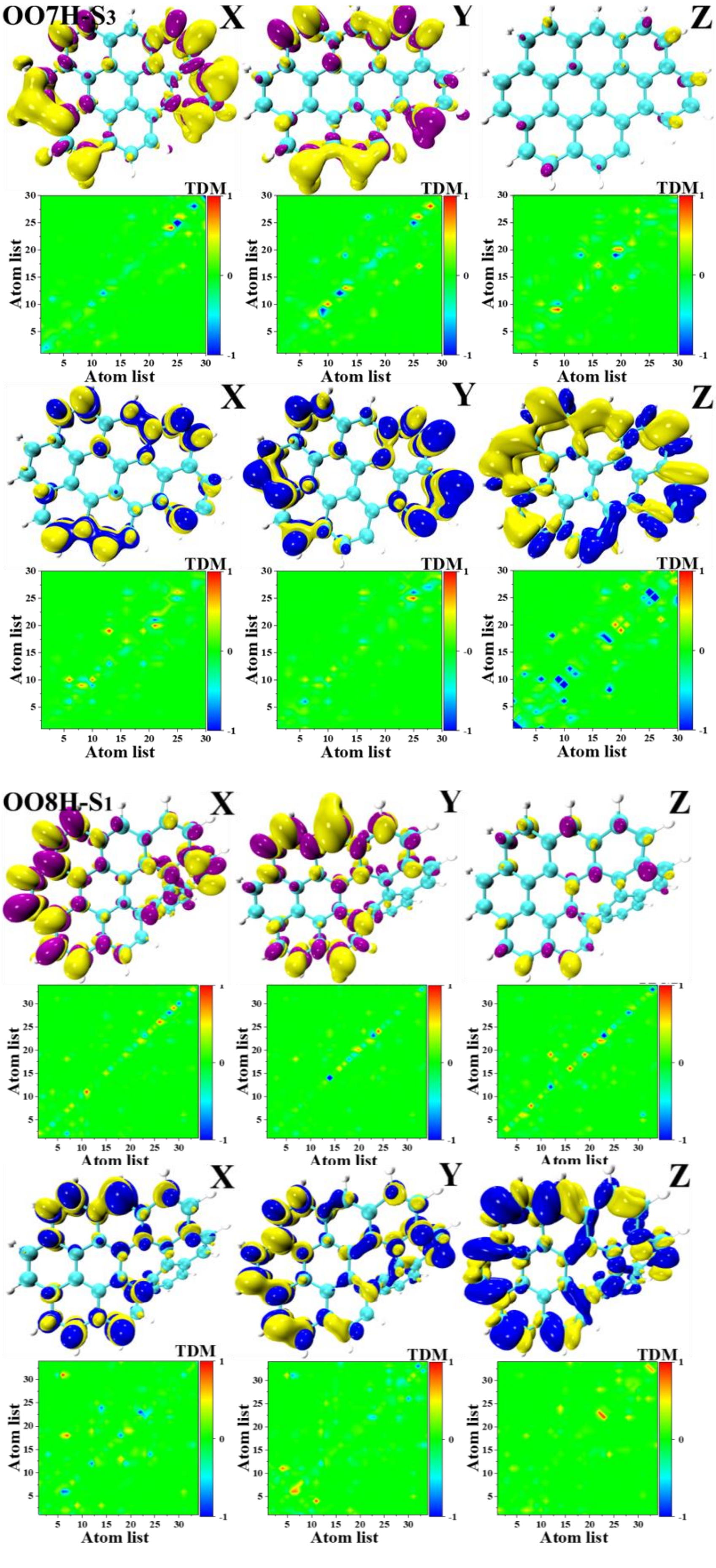

OO7H-S3
X
Y
Z
TDM
Atom list
OO8H-S1

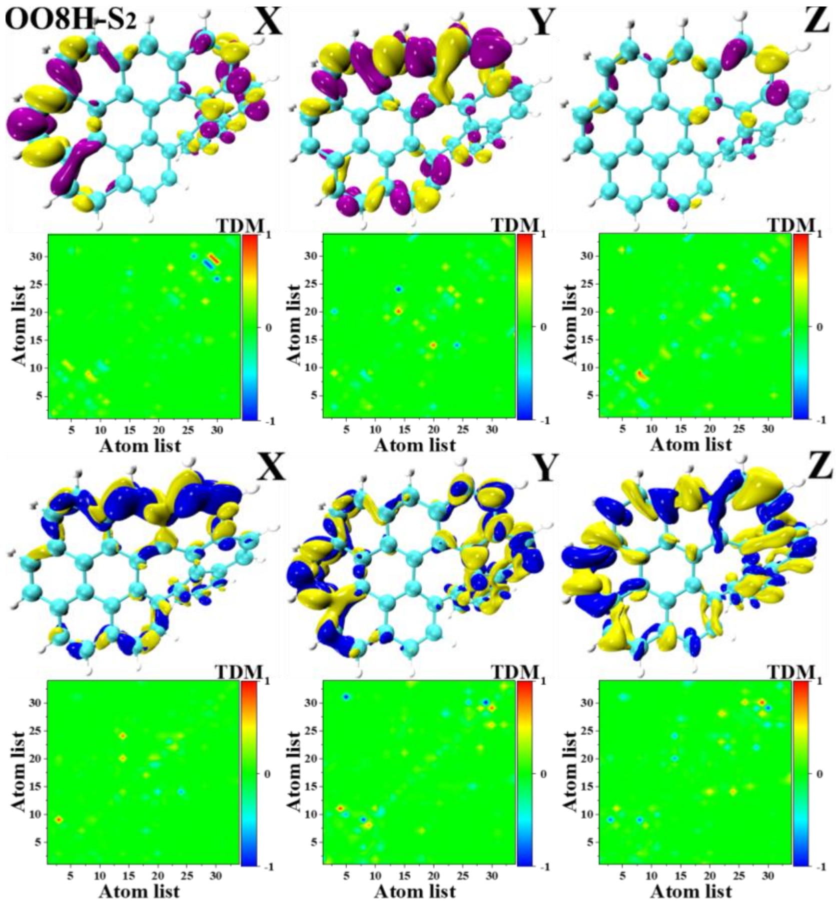
OO8H-S2
X
Y
Z
TDM
Atom list
Atom list

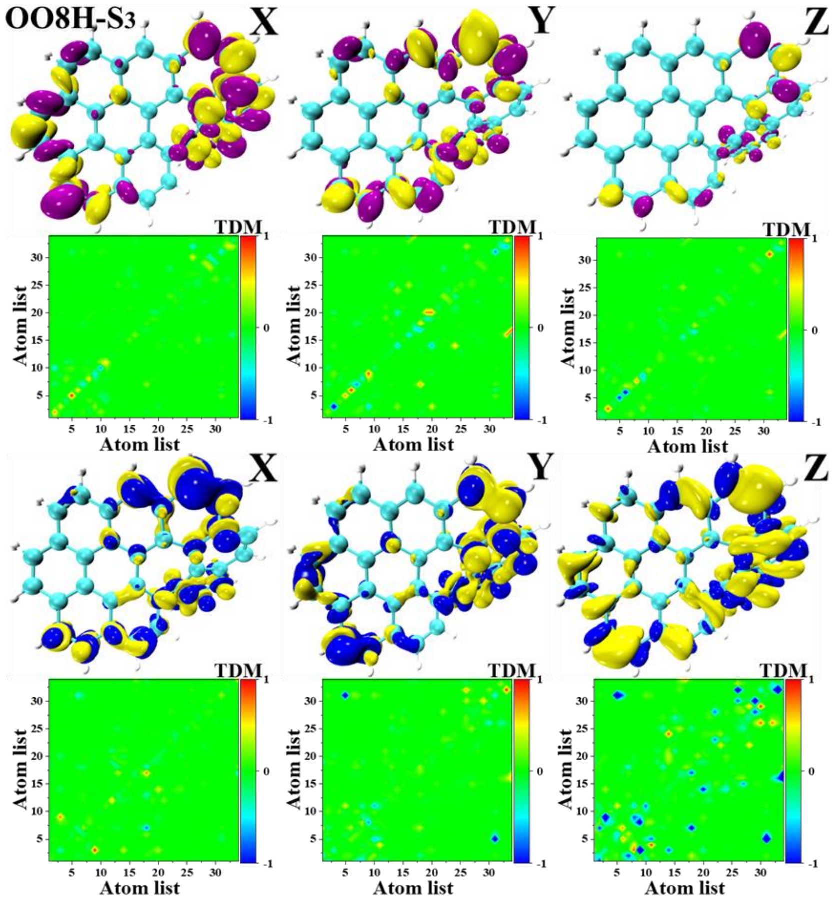
OO8H-S3
X
Y
Z
TDM
Atom list
Atom list

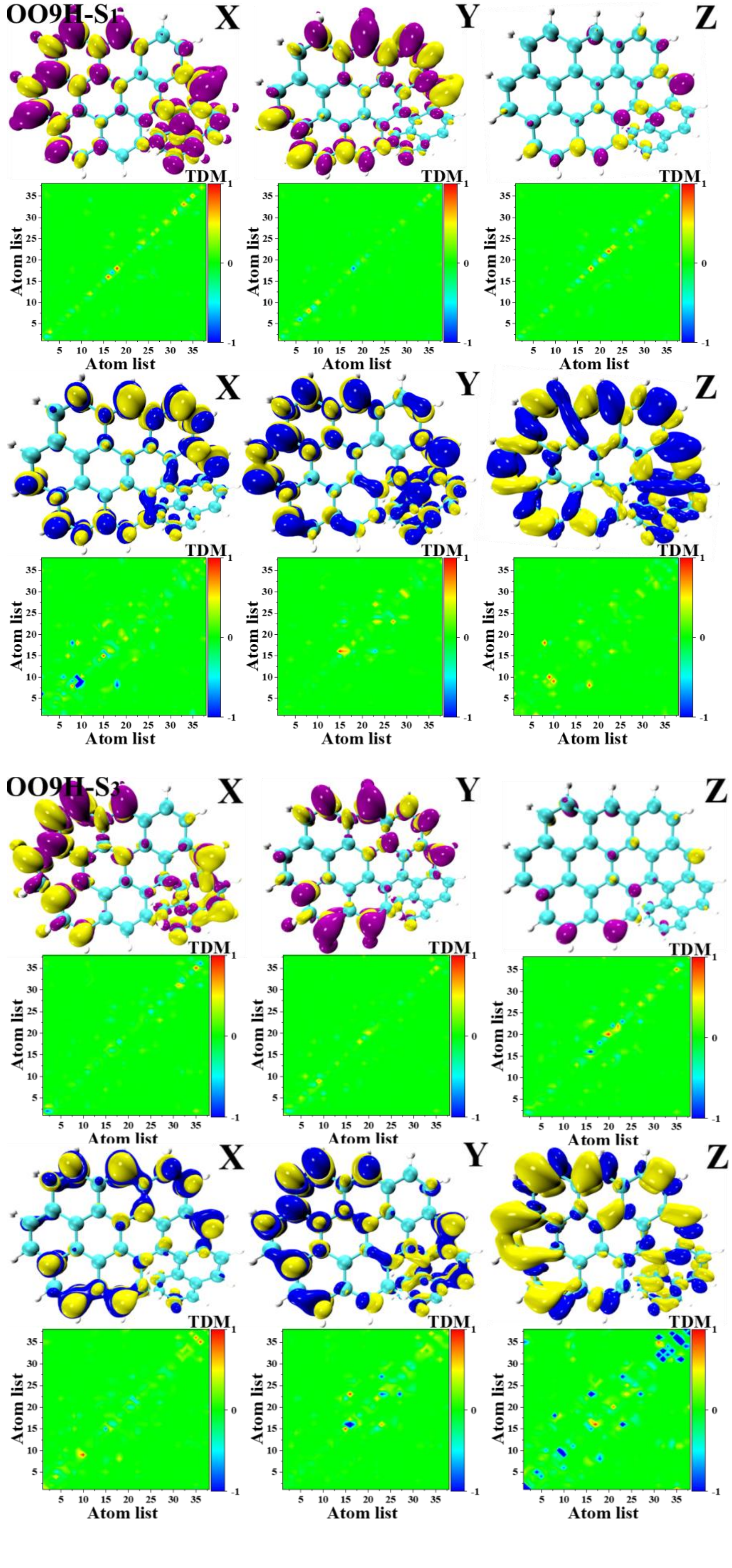
OO9H-S1
X
Y
Z
TDM
Atom list
OO9H-S2

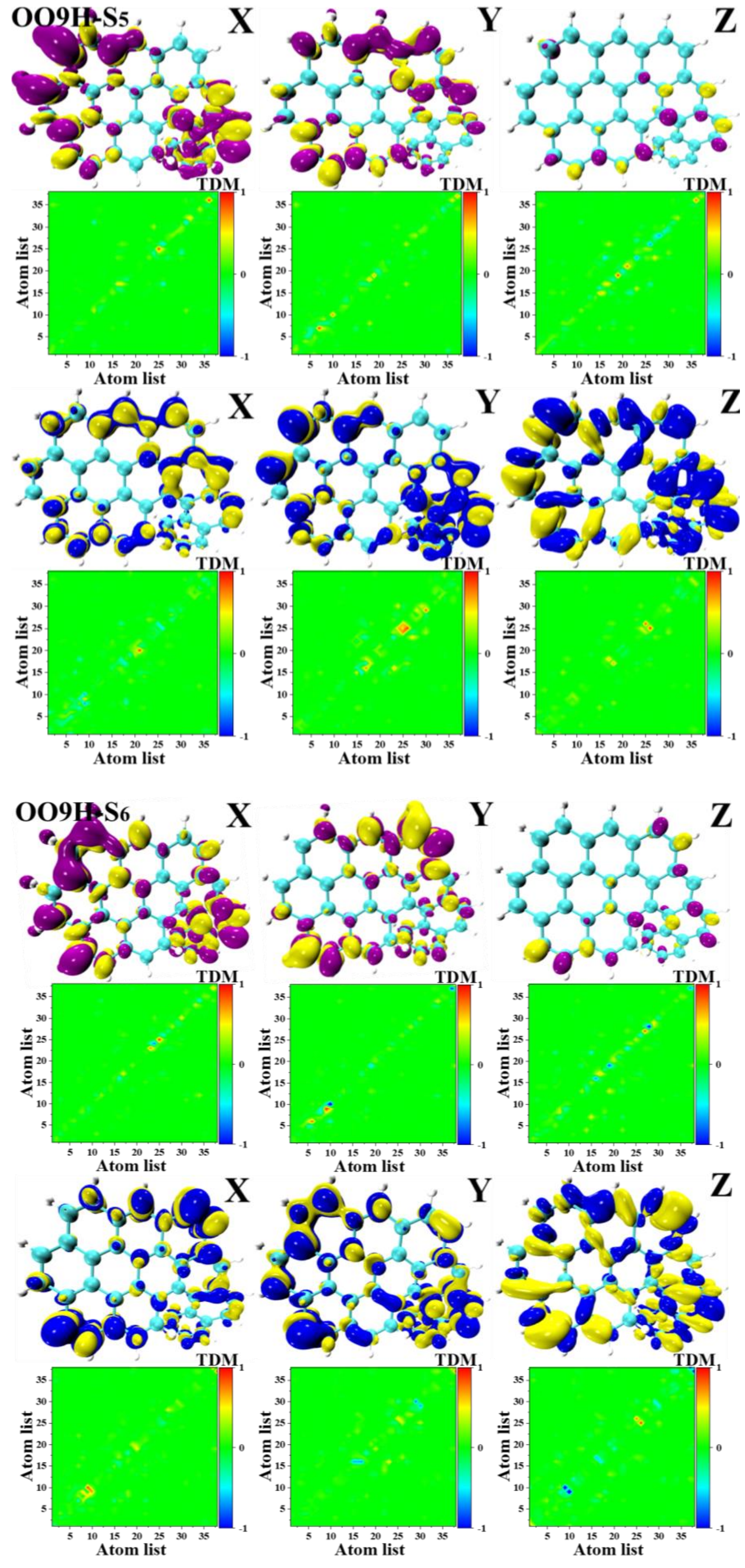

OO9H-S5
X
Y
Z
TDM
Atom list
OO9H-S6

Figure S5. 3D and 2D diagrams of transition electric couple and magnetic dipole moment of O7H, O8H, O9H, OO7H, OO8H, OO9H.

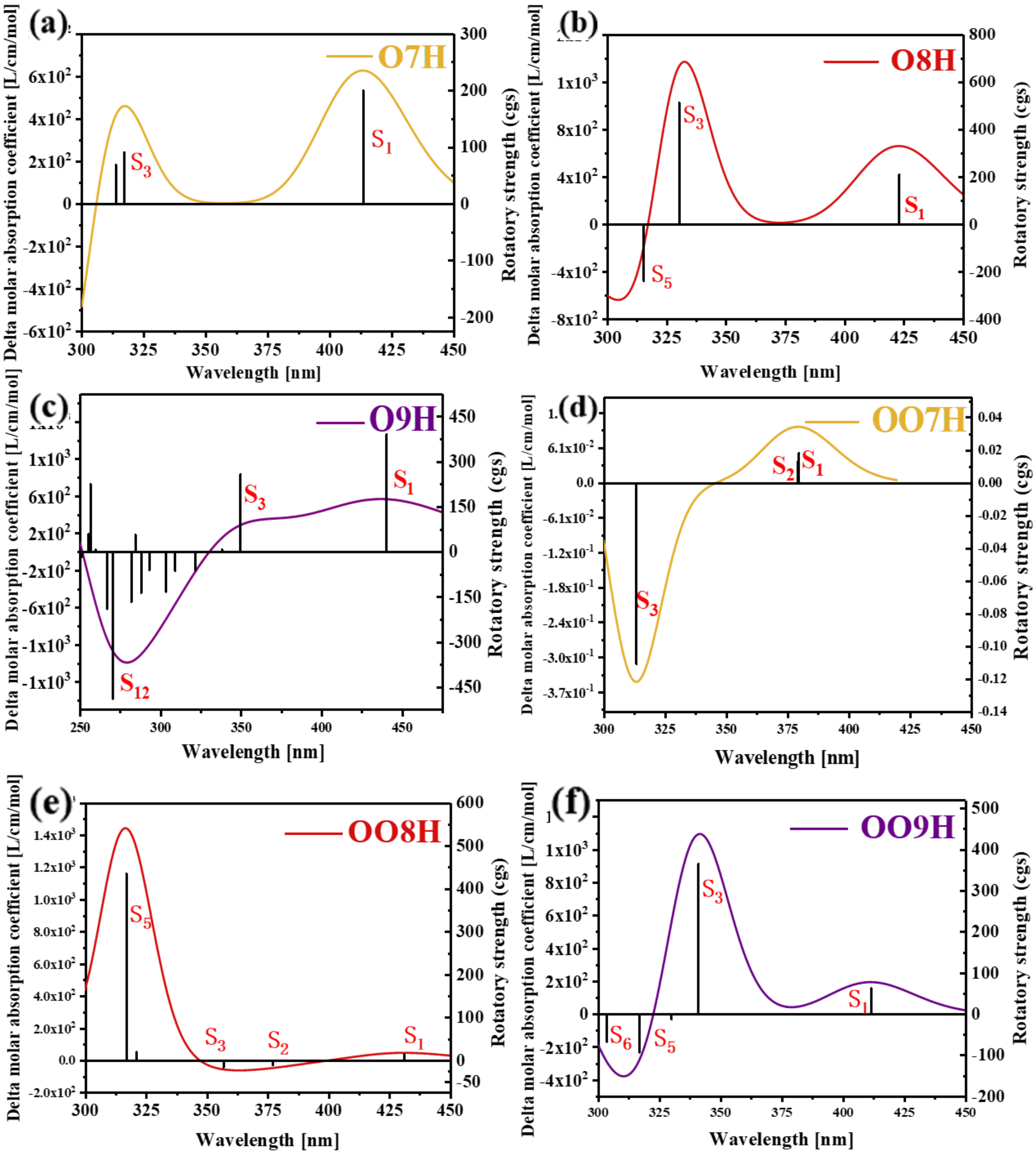

Figure S6. Electron circular dichroism of each molecule: (a) O7H, (b) O8H, (c) O9H, (d) OO7H, (e) OO8H, (f) OO9H.

(a)

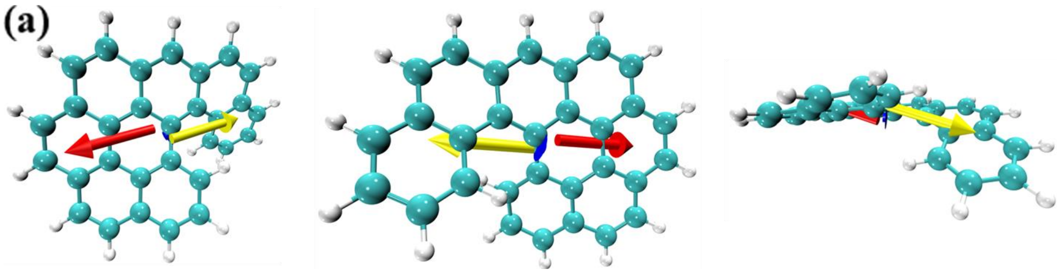

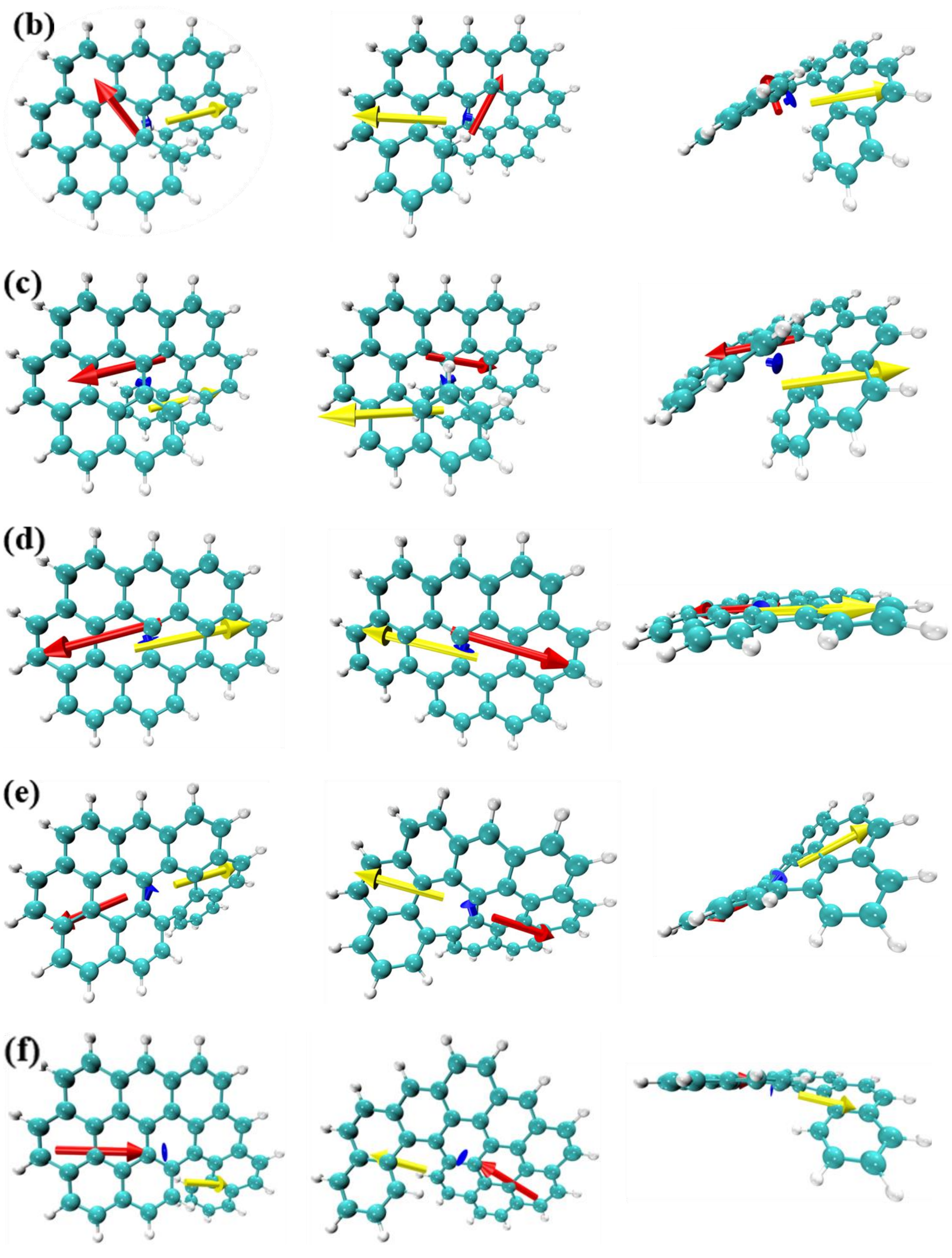

Figure S7. Front, back and bevel of dipole moments.：（a） O7H,（b） O8H,（c） O9H,（d） OO7H,（e） OO8H,（f） OO9H.
